# Interplay between MAPK signaling pathway and autophagy in skin aging: mechanistic insights and therapeutic implications

**DOI:** 10.3389/fcell.2025.1625357

**Published:** 2025-06-27

**Authors:** Xu Liu, Bo Chen, Xuefeng Liu, Xiaoqing Zhang, Jingdong Wu

**Affiliations:** ^1^ The First Clinical College, Liaoning University of Traditional Chinese Medicine, Shenyang, China; ^2^ Department of Exercise Physiology, Beijing Sport University, Beijing, China; ^3^ The Third Clinical College, Liaoning University of Traditional Chinese Medicine, Shenyang, China; ^4^ College of Acupuncture, Liaoning University of Traditional Chinese Medicine, Shenyang, China; ^5^ Department of Dermatology and Cosmetology, Liaoning University of Traditional Chinese Medicine Hospital 2nd Affiliated Hospital, Shenyang, China

**Keywords:** skin aging, MAPK, autophagy, interplay, natural bioactive compounds

## Abstract

Skin aging manifests as structural degradation, functional decline, and heightened disease susceptibility. Central to this process is the overactivation of the mitogen-activated protein kinase (MAPK) signaling pathway triggered by reactive oxygen species (ROS). Autophagy, a lysosomal degradation mechanism essential for maintaining cellular homeostasis, demonstrates context-dependent duality in skin aging by mediating cytoprotective effects and stress-induced dysfunction. Emerging evidence highlights that the interplay between MAPK signaling and autophagy critically modulates skin aging progression. Despite its therapeutic potential, the lack of effective targeting strategies severely hinders clinical translation. Therefore, this review synthesizes current evidence on MAPK–autophagy interplay across key cutaneous cell populations, namely, keratinocytes, fibroblasts, and melanocytes (including melanoma), revealing cell-type-specific regulatory networks that influence skin aging. Subsequently, we explore the therapeutic potential of natural bioactive compounds targeting this interplay to accelerate the translation of evidence into the progression of strategies for combating skin aging.

## 1 Introduction

Skin is the barrier that separates the body from the external environment and is responsible for protecting internal organs from external stimuli. As the most voluminous body organ, skin changes are the most recognizable signs of aging. Skin aging is typically characterized by thinning, dryness, reduced elasticity, and abnormal pigmentation, which is determined by the combined influence of intrinsic factors (such as gene mutations, cellular metabolism, or hormonal agents) and extrinsic factors (such as ultraviolet (UV) light, air pollution, smoking, and unhealthy diet) (Krutmann et al., 2021; Mora Huertas et al., 2016). Skin aging affects appearance and triggers health issues, including increased fragility, diminished immune function, impaired vascular support, delayed wound healing, and a heightened risk of skin malignancies. These changes ultimately lead to a decline in the structural integrity of the skin and a loss of its protective barrier function (Quan, 2023; Russell-Goldman and Murphy, 2020). With the acceleration of global aging, skin aging, particularly photoaging, has emerged as an increasingly significant health and societal concern. According to a 15-year longitudinal study, the incidence of photoaging has significantly increased from 42% to 88% (Hughes et al., 2021). Therefore, exploring effective treatments for combating skin aging and photodamage is crucial to address the health challenges of an aging society.

Multiple signaling pathways are involved in the regulation of skin aging. For instance, the transforming growth factor-β (TGF-β)/Smad pathway modulates collagen synthesis, the nuclear factor kappa B (NF-κB) pathway drives inflammatory progression, and the nuclear factor erythroid 2-related factor 2 (Nrf2) pathway mediates antioxidant responses (Chaiprasongsuk and Panich, 2022). Among these, the mitogen-activated protein kinase (MAPK) pathway has received widespread attention because of its key role in skin aging progression and related signaling pathways (Gu et al., 2020). MAPK is a serine/threonine protein kinase that converts extracellular stimuli into a wide range of cellular responses (Sun et al., 1999). Endogenous aging processes (e.g., mitochondrial dysfunction) and environmental assaults (notably UV irradiation) converge to amplify reactive oxygen species (ROS) generation, leading to oxidative stress and an overactivated MAPK signaling pathway (Giorgi et al., 2018; Zhang and Duan, 2018). The activation of this pathway results in collagen degradation, extracellular matrix (ECM) disruption, and excessive inflammatory factor release, thereby accelerating skin aging and establishing it as a key therapeutic target (Bae et al., 2008; Mantena and Katiyar, 2006; Muthusamy and Piva, 2010; Afnan et al., 2016).

Autophagy serves as a pivotal mechanism to maintain cellular homeostasis by mediating the catabolism of cytoplasmic components and the degradation and recycling of damaged proteins and organelles (Levine and Kroemer, 2019). Although autophagy activation is generally associated with delayed aging, emerging evidence suggests that it can also act as a double-edged sword in skin aging (Eckhart et al., 2019; Jeong et al., 2020b). Autophagy is closely linked to the MAPK signaling pathway, which critically regulates autophagy processes through phosphorylation events (Gu et al., 2020). However, the role of the interplay between MAPK signaling and autophagy in aging skin remains incompletely elucidated, particularly regarding cell type-specific outcomes and their therapeutic implications.

**FIGURE 1:**
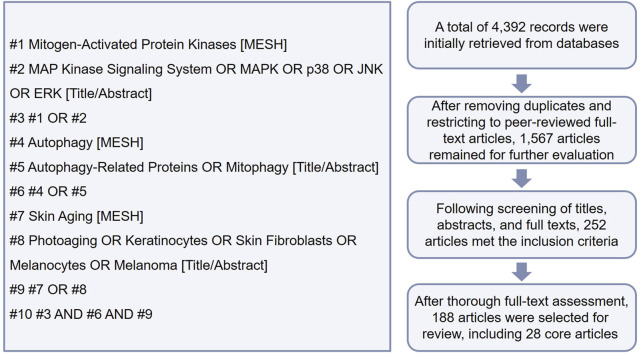
Literature search flowchart (using PubMed as an example).

In this review, 28 core articles published in recent years were collected from PubMed, Web of Science, Embase, and other databases using keywords such as “MAPK”, “autophagy”, and “skin aging” (Figure 1). The main selection criteria focused on the interplay between MAPK signaling and autophagy in regulating skin aging. This review examines the role and regulatory mechanism of the MAPK–autophagy interplay in the aging of key cutaneous cell populations, such as keratinocytes, fibroblasts, and melanocytes (including melanoma). We also explore emerging strategies targeting this interplay for anti-aging interventions, thereby providing new insights for developing effective skin anti-aging therapies.

## 2 Skin aging

**FIGURE 2:**
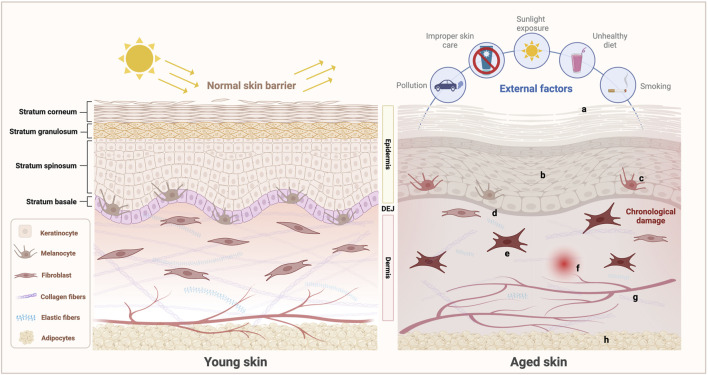
Schematic of the characteristics of young and aged skin. Under the combined influence of intrinsic and extrinsic factors, aging skin undergoes a series of complex structural and functional changes: (a) Epidermal dehydration and impaired barrier function; (b) Proliferation, differentiation, and migration abilities of keratinocytes weaken; (c) Decreased number and declined function of melanocytes, leading to abnormal melanin production and increased risk of melanoma; (d) Flattened DEJ with weakened structural integrity; (e) Senescent changes in the fibroblasts in the dermis, characterized by a reduction in number and functional decline, leading to the decreased synthesis and increased degradation of ECM components such as collagen and elastic fibers and thereby disrupting the dermal reticular structure; (f) Induced inflammation; (g) Compromised integrity of the microvascular network, resulting in vascular fragmentation; (h) Atrophied subcutaneous adipose tissue, accompanied by a reduction in adipocyte numbers. These changes collectively contribute to the characteristic manifestations of skin aging. (Created with BioRender.com).

Skin aging is a complex biological process driven by both intrinsic and extrinsic factors, ultimately leading to the progressive deterioration of skin structure and function (Figure 2). The details of these processes are elucidated in the following sections.

### 2.1 Skin structure and function

The skin, the body’s largest organ (approximately 1.8 m^2^ surface area), serves as a physical barrier against environmental pathogens/insults and a critical regulator of systemic homeostasis through water retention and thermoregulation. Its multilayered architecture and specialized cellular components collectively provide robust defense against microbial invasion, mechanical stress, and chemical exposure (Chambers and Vukmanovic-Stejic, 2020; Nestle et al., 2009). From an anatomical point of view, the skin comprises the following three distinct layers from superficial to deep: epidermis, dermis, and subcutaneous tissue. The epidermis (50–100 μm) consists of the stratum basale, stratum spinosum, stratum granulosum, and outermost stratum corneum. Keratinocytes represent the predominant cell type, constituting over 90% of epidermal cells. Other specialized cell populations include melanocytes, Langerhans cells, dendritic cells, and Merkel cells (Dahabra et al., 2021; Eckhart and Zeeuwen, 2018). Keratinocyte differentiation is initiated by proliferative activity in the stratum basale, followed by progressive migration through epidermal layers. Terminal differentiation culminates during corneocyte formation within the stratum corneum. These organelle-free dead cells establish a formidable physical barrier that effectively prevents pathogen invasion and minimizes transepidermal water loss (Nestle et al., 2009). Melanocytes, residing in the stratum basale, produce pigment-containing melanosomes that determine skin pigmentation and provide photoprotective functions through UV radiation absorption (Rittié and Fisher, 2015).

The dermis (3–5 mm), the principal structural component of the skin, lies beneath the epidermis and interfaces with it through a wavelike dermal–epidermal junction (DEJ) (Lavker et al., 1989). Histologically divided into two regions, the superficial papillary layer consists of loose connective tissues containing extensive vascular networks and sensory nerve endings that facilitate epidermal nourishment and sensory transduction. The deeper reticular layer, comprising dense irregular connective tissues rich in collagen and elastic fibers, constitutes the bulk of the dermis and confers mechanical resilience and elasticity to the skin (Brown and Krishnamurthy, 2024). The metabolically active dermis serves as the primary site for wound healing (Dahabra et al., 2021). Cellular constituents primarily include fibroblasts, macrophages, and other immune cells. Fibroblasts, the principal resident cells, synthesize and maintain collagen/elastic fibers, proteoglycans, and glycosaminoglycans, thereby establishing a 3D ECM network essential for dermal structural integrity (Gerasymchuk et al., 2020; Shin et al., 2019). These cells also play pivotal roles in wound repair and tissue remodeling (Ren et al., 2022). The dermis further harbors numerous skin appendages, including hair follicles, sebaceous glands, and sweat glands (Brown and Krishnamurthy, 2024). Beneath the dermis lies the subcutaneous tissue (hypodermis), which is predominantly composed of adipocytes, bursae, vasculature, and connective tissues. This layer provides thermal insulation, serves as an energy reservoir, and mechanically anchors the skin to underlying musculoskeletal structures while cushioning mechanical impacts (Fore, 2006).

### 2.2 Intrinsic skin aging

Intrinsic skin aging is a series of chronologically physiological changes primarily driven by genetic and hormonal factors. This process involves a cascade of molecular dysregulations, including DNA damage, telomere shortening, excessive ROS production, and mitochondrial dysfunction, which collectively drive the accumulation of senescent cells in tissues and thereby contribute to progressive senescence (Salminen et al., 2022). Age-dependent functional deterioration occurs across all skin strata—epidermis, DEJ, and dermis. Epidermal aging is characterized by diminished keratinocyte proliferative capacity, resulting in progressive thinning of both the viable epidermis and stratum corneum (Low et al., 2021). Impaired keratinocyte differentiation characterized by the reduced synthesis of keratins and structural proteins compromises the epidermal barrier integrity and hydration capacity (Wang et al., 2020). Age-related declines in keratinocyte migratory potential further disrupt reepithelialization during wound healing, resulting in delayed tissue repair (Ross et al., 2011). In addition, cellular attrition exacerbates epidermal atrophy, with melanocyte populations reportedly decreasing by 8%–20% per decade (Gilchrest et al., 1979). Intrinsic skin aging also manifests through structural alterations in the dermal papillae and basal layer, characterized by progressive thinning. This anatomical remodeling induces DEJ flattening, thereby reducing the interfacial contact area between dermal and epidermal compartments. As a consequence, the diminished exchange surface impairs nutrient diffusion to the epidermis while suppressing basal keratinocyte proliferative capacity (Russell-Goldman and Murphy, 2020; Sauermann et al., 2002).

During intrinsic aging, the dermis exhibits a progressive decline in the number and functional capacity of fibroblasts. This cellular deterioration leads to the diminished synthesis of collagen and elastic fibers, thereby contributing to cutaneous laxity and wrinkle formation (Freitas-Rodríguez et al., 2017; Kohl et al., 2011). Fibroblast senescence also contributes to skin aging by secreting a senescence-associated secretory phenotype, which decreases proliferation by impeding the release of essential growth factors and enhances the degradation of the ECM by activating matrix metalloproteinases (MMPs) (Ghosh and Capell, 2016). The ROS produced during aging are the main stimuli increasing MMP levels in aging skin, leading to collagen degradation and fibroblast senescence. Aged fibroblasts also produce additional ROS, further promoting MMP expression and forming a vicious cycle that accelerates dermal aging. This process severely hinders mechanical interactions between fibroblasts and ECM, resulting in reduced fibroblast volume (Freitas-Rodríguez et al., 2017; Shin et al., 2019). The functional decline and volume reduction of dermal fibroblasts are important reasons for the slowed wound healing in aged skin (Boismal et al., 2020).

### 2.3 Extrinsic skin aging (photoaging)

Skin aging is not solely driven by intrinsic factors; long-term exposure to solar UV radiation is the primary cause of extrinsic skin aging, also known as photoaging, which accounts for approximately 80% of total skin aging (Friedman, 2005). Photoaging and intrinsic aging overlap, causing areas frequently exposed to sunlight, such as the face, neck, forearms, and dorsal hands, to exhibit premature aging phenotypes compared with photoprotected skin (Rittié and Fisher, 2015). Photoaged skin is clinically characterized by distinct pathological features, including leathery texture, deep rhytides, telangiectasia (“broken” capillaries), mottled hyperpigmentation, and lentigines, and is in stark contrast to skin that predominantly undergoes intrinsic aging (Brooke et al., 2001). UV radiation is classified into the three subtypes based on wavelength, biological activity, and cutaneous penetration: long-wave UVA (320–400 nm), medium-wave UVB (280–320 nm), and short-wave UVC (200–280 nm) (Dorf and Maciejczyk, 2024). Despite being a weak mutagen, UVA penetrates deeply into the dermal and subcutaneous layers due to its long wavelength, acting as the principal driver of photoaging through sustained oxidative damage (Battie et al., 2014). By contrast, UVB radiation, though limited to epidermal absorption due to its short wavelengths, exhibits potent genotoxicity by directly inducing thymine dimer photoproducts that cause DNA damage, thereby initiating mutagenic processes and carcinogenesis (Guan et al., 2021). UVC demonstrates the strongest mutagenic potential but is effectively filtered by the stratospheric ozone under normal atmospheric conditions and thus fails to reach the Earth’s surface (Dorf and Maciejczyk, 2024).

UV radiation initiates a cascade of molecular and cellular events that drive accelerated skin aging. It induces ROS generation, resulting in oxidative stress and DNA damage. Concurrent inflammatory pathway activation, MMP upregulation, and disrupted collagen/elastin synthesis further exacerbate cutaneous degeneration and elasticity loss (Cai et al., 2023). A hallmark histological feature of photoaging is the accumulation of abnormally thickened and fragmented elastic fibers, termed “solar elastosis” (Tsuji, 1980). Extrinsic aging also induces a drastic reduction in type I collagen and an increase in type III collagen deposition. This imbalance in the I/III collagen ratio renders aged skin fragile and inelastic (Dorf and Maciejczyk, 2024). The epidermal rete of photoaged skin is thicker than that of chronologically aged skin, and epidermal atrophy is observed in severely photoaged skin (Bhawan et al., 1995). UV radiation-induced keratinocyte damage reduces cellular activity and decelerates epidermal turnover, leading to compromised barrier function. This effect manifests clinically as severe xerosis (dryness) and desquamation, with UV-driven effects often surpassing the severity of intrinsic aging phenotypes (Farage et al., 2013). As a photoprotective mechanism, melanogenesis is upregulated to counteract UV-generated ROS (Eller et al., 1994). However, chronic sun exposure promotes uneven pigmentation in aged skin that frequently progresses to solar lentigines, which are characteristic hyperpigmented lesions pathognomonic of photoaging (Black, 2016; Rittié and Fisher, 2015). Furthermore, excessive UV exposure increases melanoma risk through melanocytic malignant transformation, while age-related remodeling of the tumor microenvironment during physiological senescence further enhances susceptibility, establishing melanoma as a critical event in cutaneous aging (Tsai and Chien, 2022; Urban et al., 2021). Finally, photoaging accelerates the loss of subcutaneous fat, weakening the skin’s supportive structure and leading to increased sagging and hollowing of the skin (Scharffetter-Kochanek et al., 2000).

## 3 Autophagy: key event in skin aging

### 3.1 Molecular mechanisms of autophagy

Autophagy is a highly conserved cellular degradation and recycling process present in all eukaryotic organisms. In mammalian cells, autophagy primarily includes three types: chaperone-mediated autophagy (CMA), microautophagy, and macroautophagy. These types all facilitate the proteolytic degradation of cytoplasmic components within lysosomes, but their transport mechanisms differ (Mizushima and Komatsu, 2011; Yang and Klionsky, 2010). In CMA, targeted proteins are translocated across the lysosomal membrane by complexing them with chaperone proteins (such as HSC-70), which are recognized by the lysosomal membrane receptor lysosomal-associated membrane protein 2A, resulting in their unfolding and degradation (Saftig et al., 2008). In microautophagy, invaginations or protrusions of the lysosomal membrane are used to capture cargo, and the uptake occurs directly at the limiting membrane of the lysosome (Parzych and Klionsky, 2014). Macroautophagy (hereafter referred to as autophagy) is the most extensively studied type to date. It is the engulfment of large structures through selective and nonselective mechanisms and is characterized by the formation of autophagosomes. This process involves three key steps: initiation, nucleation, and elongation, each of which is regulated by multiple critical genes and signaling pathways (Cao et al., 2021). Stress signals that trigger autophagy typically encompass starvation, hypoxia, oxidative stress, protein aggregation, and endoplasmic reticulum (ER) stress (Glick et al., 2010). A classic example is starvation-induced autophagy, where a reduction in nutrient supply leads to bioenergetic stress and elevated AMP levels. This phenomenon activates AMP-activated protein kinase (AMPK), which subsequently inhibits the mammalian target of rapamycin complex 1 (mTORC1). mTORC1 inhibition promotes the assembly of the UNC-51 like kinase 1 (ULK1) complex, which comprises ULK1 (homologous to yeast autophagy-related gene 1 (ATG1)), ATG13, ATG101, and focal adhesion kinase family interacting protein of 200 kDa (FIP200). In response to stress signaling, the ULK1 complex is recruited to the phagophore assembly site on the ER, initiating autophagosome formation (Xue et al., 2024). Conversely, the presence of nutrients and growth factors activates mTORC1, leading to the phosphorylation of autophagy-related proteins and the suppression of autophagy (Kim and Guan, 2015; Wang and Zhang, 2019). In the second step, the activated ULK1 complex induces phagophore nucleation by promoting the production of phosphatidylinositol 3-phosphate (PI3P) through the formation of a supramolecular complex with class III phosphoinositide 3-kinase (PI3K) activity. This complex consists of VPS34, VPS15, ATG14, Beclin-1, autophagy and Beclin-1 regulator 1 (AMBRA1), and/or UV radiation resistance-associated gene (UVRAG). It accompanies the recruitment of ATG9-containing vesicles, and its activity is subjected to tonic inhibition by B-cell lymphoma-2 (Bcl-2). Finally, in the phagophore elongation step, ATG7 and ATG10 catalyze the formation of the ATG12–ATG5–ATG16L1 complex. At the same time, ATG4, ATG7, and ATG3 cooperate to cleave the precursor of microtubule-associated protein light chain 3 (LC3)-like proteins into their mature forms, which are then conjugated with phosphatidylethanolamine (PE) and recruited to the autophagosome with the support of WD-repeat protein interacting with phosphoinositide (WIPI) proteins. LC3 and LC3 homologs enable the autophagosome to bind to autophagy substrates and/or mediate cargo-selective proteins, including p62 (Galluzzi and Green, 2019; Xue et al., 2024). Upon completion, the phagophore is sealed to form the characteristic double-membrane autophagosome. As autophagy progresses, autophagosomes fuse with lysosomes to form autolysosomes. This fusion enables progressive acidification, consequently activating lysosomal hydrolases that mediate substrate degradation. The resulting breakdown products are released back into the cytoplasm for reuse or to provide energy (Choi et al., 2013).

### 3.2 Dual role of autophagy in skin aging

Autophagy serves as a pivotal regulator of cutaneous homeostasis, functioning as a critical mechanism of intracellular quality control for keratinocytes, fibroblasts, and melanocytes (Sukseree et al., 2013). Under physiological and stress conditions, autophagy facilitates the clearance of senescent organelles and misfolded proteins, thereby modulating skin cell functionality and delaying aging progression (Vikram et al., 2024; Wang et al., 2019b; Zhong et al., 2024). For instance, the autophagy inducer heptasodium hexacarboxymethyl dipeptide-12 (Aquatide™) enhances oxidative stress resistance in normal human epidermal keratinocytes *in vitro* while clinically improving skin elasticity and texture (Lim et al., 2019). Similarly, rapamycin (mTOR inhibitor) attenuates UVB-induced fibroblast photoaging by activating autophagy to suppress ROS accumulation (Qin et al., 2018). Preclinical studies revealed that a variety of naturally derived bioactive compounds exert significant anti-aging effects on the skin through a multitarget regulation of the autophagy pathway (Li et al., 2018; Lin et al., 2024; Yang et al., 2023). Moreover, skin aging is closely linked to and partially driven by autophagy defects (Eckhart et al., 2019). ATG7-deficient keratinocytes have exhibited increased DNA damage and senescence markers following oxidative stress induced by paraquat, a cellular senescence-inducing oxidant (Song et al., 2017). Autophagy deficiency is strongly associated with premature senescence and oxidative damage accumulation in melanocytes (Ni et al., 2016; Setaluri, 2015; Zhang et al., 2015). Furthermore, chronic and repeated UVA exposure disrupts lysosomal function in skin fibroblasts, impairing intracellular degradation mechanisms and contributing significantly to photoaging (Huang et al., 2019).

**FIGURE 3:**
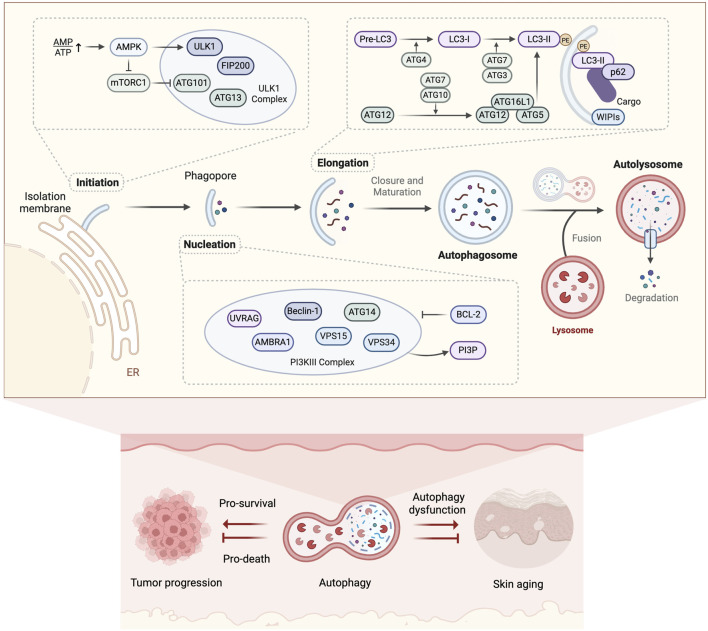
Molecular mechanisms of autophagy and its dual role in skin aging. Autophagy mediates cytosolic cargo clearance through double-membrane autophagosome formation, lysosomal degradation, and metabolite recycling, exhibiting dual regulatory roles in skin aging and associated tumor progression. (Created with BioRender.com).

Despite these compelling data, some studies took the opposite view and suggested that autophagy activation may promote skin aging. A significant increase in autophagy vesicles was observed in senescent fibroblasts (Gerland et al., 2003), and an increase in autophagic activity was reported in senescent human keratinocytes induced by ROS (Gosselin et al., 2009). These findings suggest that cells may enhance autophagic activity in response to stress to cope with damage and restore survival (protective autophagy). However, this activity may lead to the accumulation of damaged cells, thereby promoting aging. Several studies supported this view. Young et al. (2009) demonstrated that oncogene-induced fibroblast senescence depends on prior autophagic activity, and reducing autophagic expression levels through pharmacological or genetic approaches can inhibit the onset of senescence. Similarly, ATG5 overexpression has been shown to reduce the proliferation of melanoma cells and induce senescence, while autophagy inhibition delays oncogene-induced senescence (Liu et al., 2013). These results indicate that the role of autophagy in skin aging may exhibit a context-dependent duality. If autophagy is excessively activated or prolonged, it may lead to cell death due to the excessive elimination of essential cellular proteins or organelles. This phenomenon explains why autophagy is also referred to as type II programmed cell death (type I being apoptosis) (Lin et al., 2024; Tsujimoto and Shimizu, 2005). Autophagic flux-dependent outcomes have been observed in cutaneous oncology. Studies found that in senescent human keratinocytes, excessive autophagy activation can induce senescent cell death, thereby inhibiting tumors; whereas moderate autophagy activation facilitates the escape of senescent cells from death and promotes the growth of tumor cells (Deruy et al., 2014). This phenomenon has also been widely observed in melanoma (Pangilinan et al., 2024) (Figure 3). In summary, these findings position autophagy regulation as an essential therapeutic target for skin anti-aging interventions.

## 4 MAPK signaling pathway: potential bridge between skin aging and autophagy

### 4.1 Role of MAPK signaling pathway in skin aging

The signaling proteins of the MAPK pathway mainly belong to the family of serine/threonine protein kinases; this pathway is a key cellular signal transduction mechanism widely involved in the regulation of cell proliferation, differentiation, stress response, and apoptosis and other biological processes (Pearson et al., 2001). It comprises three main classes of kinases: mitogen-activated protein 3 kinase (e.g., Raf), mitogen-activated protein 2 kinase (e.g., MEK) and MAPK. Among the pathways of MAPK family, extracellular signal-regulated kinases 1 and 2 (ERK1/2), c-Jun amino-terminal kinases 1 to 3 (JNK1 to 3), and p38 MAPK (α, β, γ, and δ) are the most widely studied. By activating upstream signals through external stimuli (such as growth factors, cytokines, neurotransmitters, hormones, and cellular stress), the MAPK signaling pathway can regulate the expression of downstream target genes and thereby influence cellular physiological states (Cargnello and Roux, 2011; Gaestel, 2015).

**FIGURE 4:**
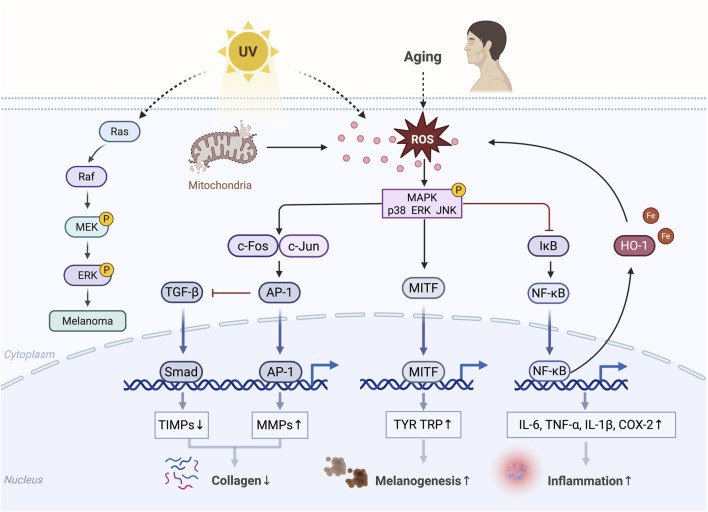
Diagram of MAPK-mediated skin aging. Phosphorylated MAPK, activated by ROS, inhibits collagen production; promotes inflammatory responses, melanogenesis, and melanoma progression; and ultimately accelerates skin aging.↑, upregulation; ↓, downregulation (Created with BioRender.com).

MAPK is important in skin aging and is primarily activated by the ROS generated in response to age-related mitochondrial dysfunction and external factors, such as UV radiation (Chaiprasongsuk and Panich, 2022; Shin et al., 2019) (Figure 4). The heterodimer activator protein 1 (AP-1) composed of c-Fos and c-Jun is a key transcription factor that regulates the expression of MMP-1, MMP-3, and MMP-9, resulting in collagen degradation. The expression of c-Jun and c-Fos is regulated by the MAPK signaling pathways, with ERK stimulating c-Fos expression; meanwhile, the activation of p38 and JNK is essential for c-Jun expression (Chiang et al., 2013; McBride and Nemer, 1998; Pramanik et al., 2003). The MAPK-induced activation of AP-1 suppresses TGF-β signaling, a key regulator of ECM biosynthesis (Gao et al., 2018; Pittayapruek et al., 2016). TGF-β enhances collagen synthesis and inhibits its degradation by downregulating MMPs through the Smad pathway and upregulating the tissue inhibitors of metalloproteinases (TIMPs) (Shin et al., 2019; Verrecchia and Mauviel, 2002). NF-κB is another important MMP transcription factor activated by the MAPK signaling (Baek et al., 2024). NF-κB activity upregulates MMPs such as MMP-1 and MMP-3 in dermal fibroblasts (Lee et al., 2012; Park et al., 2014). In addition, ROS and activated MAPK signaling pathways facilitate the dissociation of the NF-κB inhibitor protein IκB in the cytoplasm, leading to the translocation of NF-κB into the nucleus where it induces the expression of pro-inflammatory cytokines, such as tumor necrosis factor-α (TNF-α), interleukin-6 (IL-6), IL-1β, and cyclooxygenase-2 (COX-2), contributing to skin damage and inflammatory responses (Bell et al., 2003; Han et al., 2022; Wang et al., 2019a). These pro-inflammatory cytokines could further induce the expression of MMPs (Nisar et al., 2022). Activated NF-κB upregulates the expression of heme oxygenase-1 (HO-1), indirectly increasing the levels of free iron in the cells and thereby promoting ROS production through the Fenton reaction (Kammeyer and Luiten, 2015; Li et al., 2019). The MAPK signaling can also mediate UVB-induced melanogenesis (Hu et al., 2019; Zhou et al., 2022). Its abnormal activation is a major cause of melanoma progression. For instance, UV radiation triggers the sequential activation of Ras, Raf, MEK, and ERK in cells, thereby regulating various carcinogenic biological activities (Guo et al., 2021).

### 4.2 Role of MAPK and autophagy in skin aging

**FIGURE 5:**
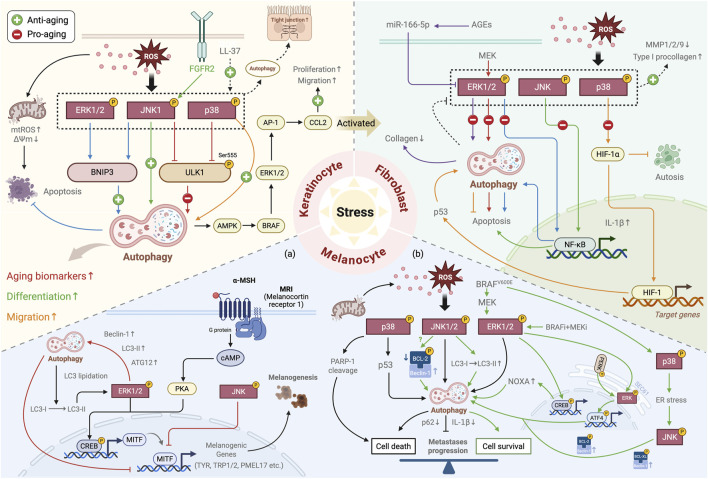
Summary of the role of the MAPK–autophagy interplay in skin aging. Under stress conditions, the interplay between the MAPK signaling and autophagy plays a crucial regulatory role in key skin cell populations: in keratinocytes, it regulates cell proliferation, differentiation, and migration, influencing epidermal barrier function and the expression of aging-related biomarkers; in skin fibroblasts, it primarily modulates the crosstalk between autophagy and apoptosis and collagen synthesis and degradation, participating in skin aging; in melanocytes **(a)** it regulates melanogenesis (black pathway) and degradation (red pathway); and in melanoma **(b)** it exhibits pro-tumor (green pathway) and anti-tumor (black pathway) effects by modulating the balance between cell death and survival. In the diagram, the differently colored lines illustrate the regulatory mechanisms mediated by the interplay of specific MAPK subtypes and autophagy, and the dotted lines represent the overall activation/inhibition of the MAPK pathway.↑, upregulation; ↓, downregulation (Created with BioRender.com).

Increasing evidence highlights a mechanistic interplay between the MAPK signaling pathway and autophagy that modulates the aging of key skin cell populations, such as keratinocytes, fibroblasts, and melanocytes (including melanoma) (Figure 5; Table 1). The specific mechanisms involved are detailed in the following subsections.

**TABLE 1:** The regulatory role of MAPK–autophagy interplay in skin aging.

Skin cell types	Functional module	Mechanisms and involved molecules	References
Keratinocyte	Photoprotection	• MAPK/ULK1-mediated autophagy regulation • BNIP3-dependent autophagy activation	Gu et al. (2022b), Gu et al. (2022a) and Moriyama et al. (2017)
	• Promotes cell migration, differentiation and proliferation • Enhances epidermal barrier function	• BNIP3-dependent autophagy activation • Interaction between p38 MAPK and ATG5 • FGFR2b/JNK-induced autophagy activation • Membrane distribution of tight junction proteins • Upregulation of chemokine CCL2	Ikutama et al. (2023), Li et al. (2019), Nanni et al. (2018), Qiang et al. (2020) and Zhang et al. (2019)
Fibroblast	Modulates collagen synthesis and influences wound healing progression	• Expression of MiR-106b-5p in endothelium-derived exosomes • Upregulation of chemokine CCL2 • Upregulation of type I procollagen • Downregulation of MMPs	Lim et al. (2020), Qiang et al. (2020) and Zeng et al. (2019)
	Regulates autophagy-apoptosis crosstalk	• NF-κB-dependent autophagic cell death • MEK/ERK-induced pro-apoptotic autophagy • HIF-1α/p53 axis modulation	Lim et al. (2021), Bravo-San Pedro et al. (2013) and Zuo and Ma (2024)
Melanocyte	Regulates melanin production	• Expression of melanogenesis-related factors (MITF, TYR, PMEL17, TRP1/2)	Cho et al. (2017) and Yun et al. (2016)
	Anti-melanoma	• MAPK-mediated autophagic cell death • Downregulation of p62 and IL-1β	Liu et al. (2009), Cho et al. (2017) and Tan et al. (2018)
	Pro-melanoma	• NOXA-dependent autophagy activation • MAPK-mediated protective autophagy • ER-mediated protective autophagy	Liu et al. (2014), Jeon et al. (2023), Hseu et al. (2019), Valli et al. (2020), Corazzari et al. (2015) and Ojha et al. (2019)

#### 4.2.1 Role of MAPK–autophagy interplay in keratinocytes

Keratinocytes are central to skin aging due to their functional decline and diminished regenerative capacity, which compromise epidermal barrier integrity and accelerate aging-related pathologies (Jeong et al., 2020b). The MAPK signaling pathway serves as a crucial regulator of autophagy and aging mechanisms in these cells. Pharmacological inhibition of the p38 MAPK pathway activates autophagy, effectively counteracting oxidative stress-induced senescence in HaCaT keratinocytes and mitigating UVB-induced photoaging in murine models. This phenomenon is exemplified by the p38 MAPK activator dehydrocorydaline (DE), which suppresses autophagy by elevating p62 expression and reducing Beclin-1 activation alongside diminished LC3II/I conversion. Conversely, the p38 MAPK inhibitor SB203580 amplifies autophagic activity (Gu et al., 2022b). Parallel research revealed that the dual inhibition of p38 MAPK and JNK pathways enhances autophagy in HaCaT cells exposed to the free radical generator AAPH, thereby diminishing the expression of senescence markers p21, p16, and K9-trimethylated histone 3 (K9M-H3) and concurrently ameliorating UVB-induced photodamage *in vivo*. These effects are mediated by the phosphorylation of ULK1 at Ser555, with the MAPK modulation directly influencing ULK1 phosphorylation status and subsequent autophagic activity (Gu et al., 2022a). This result reveals that autophagy regulation in keratinocytes depends on MAPK signaling, with ULK1 serving as its essential downstream mediator. Previous studies in other cell types have demonstrated that the MAPK/ULK1 axis critically modulates autophagy-related inflammatory responses and aging (Jin et al., 2022; She et al., 2018; Slobodnyuk et al., 2019). All these findings highlight MAPK pathway inhibition as a promising therapeutic strategy against keratinocyte senescence through autophagy activation. Further exploration of MAPK/ULK1 axis regulatory mechanisms remains essential for advancing cutaneous anti-aging interventions.

Contrary to the above findings, MAPK inactivation suppresses autophagic flux in psoriasiform keratinocytes (Wang et al., 2021), suggesting the context-dependent pro-autophagic roles of MAPK activation. UVB irradiation upregulates BCL2 and adenovirus E1B 19-kDa interacting protein 3 (BNIP3), a pro-autophagy mitochondrial protein, protecting human primary epidermal keratinocytes from apoptosis (Moriyama et al., 2014). Further studies revealed that BNIP3-induced autophagy occurs through the UVB-generated ROS-mediated activation of JNK and ERK, wherein JNK regulates and synergizes with ERK1/2 to enhance BNIP3 expression. This autophagic process increases mitochondrial membrane potential, reduces mitochondrial ROS production, and degrades dysfunctional mitochondria, ultimately protecting keratinocytes from apoptosis (Moriyama et al., 2017). These findings suggest that MAPK-mediated autophagy is crucial for shielding keratinocytes from UV-induced photodamage. Furthermore, the interplay between MAPK signaling and autophagy critically regulates keratinocyte migration, differentiation, and proliferation. During wound healing, the ROS accumulation induced by the hypoxic microenvironment enhances the phosphorylation of p38 MAPK and JNK, which in turn upregulates BNIP3-mediated autophagy and facilitates HaCaT cell migration (Zhang et al., 2019). However, a high-glucose environment inhibits the p38 MAPK pathway, leading to autophagy inactivation and blocked HaCaT cell migration; the p38 MAPK activator MKK6(Glu) can restore migratory capacity in an autophagy-dependent manner. Immunoprecipitation experiments showed that p38 MAPK–ATG5 interaction is strengthened by MKK6(Glu) overexpression but weakened upon ATG5 silencing, suggesting that the p38 MAPK activates autophagy through the transcriptional regulation of ATGs to promote cell migration (Li et al., 2019). Specifically expressed in epithelial cells, fibroblast growth factor receptor 2b (FGFR2b) can induce the transcriptional activation of autophagy by triggering the downstream JNK1 pathway, thereby promoting early differentiation in HaCaT cells (Nanni et al., 2018). Recent studies in human primary epidermal keratinocytes and their engineered 3D skin equivalent models showed that cathelicidin LL-37, a multifunctional antimicrobial peptide expressed in keratinocytes, induces the phosphorylation of ERK, JNK, and p38 MAPK, triggering autophagy. This process enhances cellular differentiation, promotes the membrane distribution of tight junction proteins such as Claudin-1 and ZO-1, and ultimately improves epidermal barrier function (Ikutama et al., 2023). All these results suggest that the modulation of autophagy by the MAPK pathway (either activation or inhibition) and its subsequent effects on keratinocyte senescence are context-dependent, varying with stress conditions and cell model. For example, in the same cell model, oxidative stress activates p38 MAPK to suppress autophagy and accelerate senescence (Gu et al., 2022b), whereas a high-glucose environment inhibits p38 MAPK, impairing autophagy and cell migration (Li et al., 2019). Under oxidative stress, JNK suppresses autophagy in immortalized HaCaT cells but promotes it in primary keratinocytes (Gu et al., 2022a; Moriyama et al., 2017). These cellular context differences may explain the subtype-specific effects of MAPKs. Notably, the interplay between MAPK signaling and autophagy is binary, and the autophagy can regulate MAPK activity reversely. Qiang et al. (2020) demonstrated that autophagic processes activate AMPK/B-Raf proto-oncogene (BRAF)/ERK1/2/AP-1 signaling cascade, thereby enhancing chemokine C-C motif ligand 2 (CCL2)-mediated keratinocyte migration and proliferation. This intricate bidirectional regulatory network underscores the critical role of MAPK–autophagy interplay in keratinocyte senescence. Thus, the mechanistic link between MAPK signaling pathway and autophagy in keratinocytes deserves further exploration.

#### 4.2.2 Role of MAPK–autophagy interplay in skin fibroblasts

The interplay between MAPK signaling and autophagy plays a pivotal role in skin fibroblast senescence. Although early investigations revealed comparable autophagic flux between aged and young fibroblasts, autophagy may not be sufficient to maintain cellular cleanliness due to the increased accumulation of waste products resulting from the increased metabolic rate of senescent cells, which can lead to skin aging (Kim et al., 2018). Recent studies have positioned fibroblast autophagy as a critical determinant in skin rejuvenation (Lu et al., 2024), with the combined regulation of MAPK and autophagy demonstrating protective efficacy against UVB-induced photoaging (Kim et al., 2024; Wen et al., 2018; Zhang et al., 2022). Primarily produced in the dermis, advanced glycation end products (AGEs) are critical mediators of skin aging (Chen et al., 2020). MiR-106b-5p in endothelium-derived exosomes induced by AGEs targets and downregulates ERK1/2, which in turn activates autophagy in human primary foreskin fibroblasts, leading to the reduction of collagen and finally resulted in the delayed wound healing (Zeng et al., 2019). However, in a full-thickness excisional wound mouse model, autophagy upregulates CCL2 expression via the AMPK/BRAF/ERK1/2/AP1 pathway, enhancing fibroblast activation and coordinating keratinocyte-fibroblast interactions to facilitate wound healing (Qiang et al., 2020). Furthermore, activating autophagy in HDFs reduces ROS-mediated MAPK activation under UVB irradiation, leading to a decreased expression of MMP-1, MMP-2, and MMP-9 and a promoted production of type I procollagen (Lim et al., 2020). These results further highlight the context-dependent interplay between MAPK signaling and autophagy in skin aging regulation. Interestingly, an increase in BNIP3 levels in dermal fibroblasts was also observed during wound healing under hypoxic conditions, further activating the autophagic process and promoting cell migration and proliferation (Zhang et al., 2024). Whether BNIP3 upregulation in skin fibroblasts is regulated by MAPK signaling pathways, as observed in keratinocytes, requires further confirmation in future studies.

The crosstalk between autophagy and apoptosis in fibroblasts, governed by MAPK signaling, represents a critical regulatory axis in cutaneous aging. Apoptosis plays a pivotal role in aging, and the accumulation of apoptotic cells accelerates skin aging (Guerrero-Navarro et al., 2024; Victorelli et al., 2023). Mechanistic studies revealed that ROS/ERK/NF-κB pathway drives autophagy-dependent apoptosis in HDFs exposed to bisphenol A (a chemical known to accelerate skin aging), significantly compromising cellular viability. Pharmacological inhibition of ERK signaling attenuates autophagic gene expression and delays autophagic cell death (Lim et al., 2021). Despite parallel investigations demonstrating the ROS/JNK/NF-κB-mediated induction of apoptosis and IL-1β secretion in HDFs, the involvement of autophagy in this pathway requires further validation (Park et al., 2021). In dermal fibroblasts derived from patients with Parkinson’s disease, the autophagy induced by the MEK/ERK1/2 pathway enhances cellular sensitivity to damage and leads to increased apoptosis, which can be reversed through MAPK pathway inhibition (Bravo-San Pedro et al., 2013). These findings suggest that inhibition of MAPK-mediated pro-apoptotic autophagy may delay skin aging under different pathological conditions. However, in cases of autophagy dysregulation (such as excessive protective autophagy), inducing apoptosis is crucial for eliminating the accumulation of senescent or damaged cells. Previous work showed that autophagy is required for UVB-induced aging in HDFs, with its inhibition redirecting cell fate from senescence to apoptosis clearance (Cavinato et al., 2017). Lu et al. (2024) further proposed that prolonged autophagy in fibroblasts impedes harmful substance clearance. In this situation, inhibiting autophagy and promoting fibroblast apoptosis can help alleviate the negative state of the skin, leading to the generation of fibroblasts that secrete fibrous and amorphous ECM proteins and thereby maintaining the integrity of the skin structure. Recent studies have found that regulating the MAPK signaling pathway can trigger a shift from protective autophagy to apoptosis in human skin hypertrophic scar fibroblasts. Specifically, inhibiting ROS-mediated p38 MAPK phosphorylation under hypoxic and ischemic conditions can decrease the expression levels of hypoxia-inducible factor-1α (HIF-1α) and p53, leading to a reduction in LC3-II and Beclin-1. Meanwhile, the expression levels of cleaved caspase 3 and Bax increases, and autosis (a form of autophagy-dependent non-apoptotic cell death) is observed (Zuo and Ma, 2024). All these findings suggest that modulating the MAPK signaling pathway could be a promising strategy to balance autophagy and apoptosis for anti-aging interventions. Therefore, exploring the modulation of the MAPK signaling pathway under different conditions to effectively manage the transition between autophagy and apoptosis in skin fibroblasts and achieve anti-aging effects deserves further investigation.

#### 4.2.3 Role of MAPK–autophagy interplay in melanocytes and melanoma

##### 4.2.3.1 Melanocytes

Pigmentation changes during aging; hyperpigmentation, which is characterized by increased melanin production, affects the skin’s appearance, impairs its barrier function, and exacerbates skin aging (Kovacs et al., 2022; Lee, 2021). Emerging evidence positions autophagy-related proteins and MAPK signaling as coregulators of melanogenic pathways (Chen et al., 2021; Jeong et al., 2020a; Jeong et al., 2019; Lee et al., 2022; Yu et al., 2024; Zhu et al., 2020), though their mechanistic interplay remains elusive. Central to this process is microphthalmia transcription factor (MITF), the master regulator of melanocyte differentiation (E, 2017). In Melan-a melanocytes, knockdown of LC3 (but not Beclin-1/ATG5) significantly reduces MITF expression and melanogenesis. Specifically, LC3 knockdown suppresses α-melanocyte-stimulating hormone (α-MSH, a representative stimulator of MITF expression)-mediated melanogenesis by attenuating cAMP response element-binding protein (CREB) phosphorylation and MITF expression via decreased ERK activity, thereby downregulating melanogenesis-related genes such as tyrosinase (TYR) and premelanosome protein (PMEL) 17. ERK overexpression reverses the effect of LC3 knockdown on CREB phosphorylation and MITF expression (Yun et al., 2016). In contrast to normal melanocytes, ERK1/2 activation in melanoma cells induces the autophagic degradation of MITF via Beclin-1/ATG12/LC3-II upregulation, leading to the downregulation of TYR and tyrosinase-related proteins (TRP) 1/2 and ultimately suppressing melanin production (Cho et al., 2017). Moreover, MAPK and autophagy may regulate melanin homeostasis in different cellular contexts. A study found that in normal melanocytes, the activation of the JNK pathway inhibits MITF expression, exerting an antimelanogenic effect; under H_2_O_2_-induced oxidative stress, autophagy activation sustains MITF expression to promote cell survival (Cho, 2021). Unfortunately, this work did not explore the specific relationship between the MAPK signaling pathway and autophagy. These results suggest that the interplay between MAPK signaling and autophagy bidirectionally regulates melanogenesis, with the direction of regulation dependent on the cellular context (physiological or pathological conditions). Considering the important therapeutic potential of the MAPK signaling pathway and autophagy in melanogenesis and age-related hyperpigmentation, further research is needed to elucidate their interplay mechanisms.

##### 4.2.3.2 Melanoma

Melanoma, a highly invasive malignant tumor resulting from melanocyte transformation, represents a key pathological event associated with skin aging. Approximately 60% of melanomas contain BRAF proto-oncogene mutations, with the most common being the valine-to-glutamic acid substitution at codon 600 (BRAF^V600E^), which leads to the abnormal activation of MAPK signaling (Davies et al., 2002; Holderfield et al., 2014). Autophagy is pivotal in melanoma progression through its intricate interplay with MAPK signaling. For instance, mitochondrial-derived ROS engages p38 MAPK/p53 signaling to induce pro-apoptotic autophagy in human melanoma cells (Liu et al., 2009). ERK1/2-mediated autophagic cell death, induced by immune modulators, enhances radiotherapy sensitivity and anti-tumor immunity in preclinical mouse models (Cho et al., 2017). In addition, inducing autophagy through the simultaneous inhibition of p38 MAPK and activation of JNK1/2 reduces p62 levels and IL-1β secretion, effectively inhibiting metastatic growth in melanoma cells (Tan et al., 2018). These results indicate that activating autophagy via MAPK signaling may positively influence melanoma treatment outcomes. However, accumulating evidence suggests that autophagy may also contribute to the progression of drug resistance in melanoma (Bahar et al., 2023; Fratta et al., 2023; Pangilinan et al., 2024). BRAF^V600E^-mediated MEK/ERK/CREB signaling upregulates NOXA to trigger autophagy, enabling melanoma cells to acquire anti-apoptotic capabilities under nutrient starvation conditions (Liu et al., 2014). Photodynamic therapy-mediated MAPK activation exemplifies pathway-specific outcomes: p38 MAPK promotes apoptosis by upregulating cleaved poly (ADP-ribose) polymerase (PARP) 1, and JNK1/2 facilitates autophagosome formation through LC3 conversion, thereby protecting melanoma cells from death. JNK1/2 may also regulate Bcl-2 phosphorylation to release Beclin-1 for autophagy induction, as indicated by the observed increase in Beclin-1 levels and decrease in Bcl-2 levels (Valli et al., 2020). ER-mediated protective autophagy represents another critical resistance mechanism in BRAF-mutant melanoma (Ma et al., 2014). BRAF^V600E^ induces chronic ER stress by activating the p38 MAPK signaling pathway, which in turn activates the JNK signaling pathway. Activated JNK releases Beclin-1 by phosphorylating Bcl-2 and B-cell lymphoma-extra large (Bcl-XL), thereby activating autophagy and enhancing cellular resistance to apoptosis in melanoma cells (Corazzari et al., 2015). Following BRAF and MEK inhibition (BRAFi + MEKi) treatment in BRAF mutant melanoma, the MAPK signaling pathway translocates into the ER via an SEC61-dependent mechanism. ER-localized ERK is rephosphorylated by protein kinase R-like endoplasmic reticulum kinase (PERK), leading to the reactivation of ERK. This reactivated ERK further phosphorylates activating transcription factor 4 (ATF4), thereby triggering cytoprotective autophagy (Ojha et al., 2019). These results collectively indicate that the regulation of melanoma cell death depends on the balance between pro-death and pro-survival mechanisms. The dual role of MAPK-mediated autophagy could be attributed to autophagic flux regulation. Although autophagy regulates multiple cell death modalities, it is typically initiated as a cytoprotective mechanism under conditions of low basal autophagy, such as nutrient starvation or ER stress. However, excessive autophagic flux, usually induced by pharmacological treatment or specific therapies, can trigger autophagic cell death and exert anti-tumor effects (Liu et al., 2023). Therefore, targeting the MAPK pathway to modulate autophagic flux from pro-survival to pro-death offers a promising strategy against melanoma drug resistance, warranting further investigation.

## 5 MAPK–autophagy axis modulation by natural bioactive compounds in skin aging

Natural bioactive compounds from plants and other natural sources have been widely used to combat skin aging due to their excellent photoprotective, antioxidant, and low-risk (or no-risk) properties (He et al., 2024; Hu et al., 2024; Tanveer et al., 2023). These compounds influence skin aging through multifaceted mechanisms, with growing evidence highlighting their capacity to regulate autophagy via MAPK pathway manipulation (Table 2). Bamboo leaf flavonoids can activate autophagy by inhibiting the p38 MAPK signaling pathway, thereby reducing oxidative stress-induced keratinocyte senescence and ultimately alleviating UVB-induced photoaging in mice (Gu et al., 2022b). Another natural flavonoid compound found in *Angelica sinensis*, 4,4ʹ-dimethoxychalcone, can also activate autophagy by inhibiting the p38 MAPK and JNK signaling pathways, showing photoprotective effects *in vitro* and *in vivo* (Gu et al., 2022a). Novel drug delivery systems may allow for the improved absorption and skin anti-aging activity of these compounds (Tavakoli and Klar, 2020). In murine full-thickness skin defect models, a temperature-sensitive hydrogel loaded with taxifolin (a flavonoid derived from Siberian larch) accelerates wound healing by activating the MAPK signaling pathway, which downregulates p62 and upregulates the expression of autophagy-related proteins (LC3-II, Beclin-1, ATG5, and ATG7) (Ding et al., 2023). However, in the same delivery system and *in vivo* model, ginsenoside Rg3 (an important components from the traditional Chinese medicine Panax ginseng) promotes wound healing by increasing the expression of autophagy proteins through the inhibition of the MAPK and NF-kB pathways (Peng et al., 2022). Another traditional Chinese medicine, resveratrol-loaded mesoporous silica nanoparticles transition skin hypertrophic scar fibroblasts from protective autophagy to apoptosis by suppressing the ROS/p38 MAPK/HIF-1α/p53 signaling axis, thereby inhibiting hypertrophic scar formation (Zuo and Ma, 2024). Furthermore, dietary exogenous nucleotides improve mitochondrial function and reduce senescence marker p16 expression in senescence-accelerated mouse prone-8 mice by activating autophagy via MAPK pathway inhibition and AMPK pathway activation (Fan et al., 2024). The natural carotenoid astaxanthin reverses bisphenol A-induced autophagic cell death in HDFs by suppressing the ROS-ERK-NF-κB signaling axis (Lim et al., 2021). These findings reveal that natural bioactive compounds exhibit multi-target and multi-pathway mechanisms against skin aging through their diverse classes or optimized delivery system-based combinatorial strategies. Anticancer applications include *Polygonatum cyrtonema* lectin, extracted from the *Polygonatum cyrtonema* plant, which induces autophagic cell death in human melanoma A375 cells through the mitochondria-mediated ROS/p38/p53 pathway (Liu et al., 2009). In a lung metastasis model established through the tail vein injection of B16F10 melanoma cells in C57BL/6 mice showed that algal oil rich in n-3 polyunsaturated fatty acids effectively inhibits the metastatic outgrowth of melanoma cells by inactivating p38 MAPK and activating JNK1/2 to induce autophagy (Tan et al., 2018). However, several natural bioactive compounds, such as amide alkaloid piperlongumine and bufadienolide derivative kalantuboside B, simultaneously trigger ERK-dependent apoptosis and protective autophagy in human melanoma cells (Hseu et al., 2019; Jeon et al., 2023). This dual effect may arise from differences among natural compound classes and the roles of specific MAPK subtypes, wherein sustained ERK activation and its interplay with autophagy established as a key mechanism underlying acquired resistance in multiple tumor types (Bahar et al., 2023; Huang et al., 2023). Notably, inhibiting ERK-mediated autophagy enhances anticancer efficacy (Jeon et al., 2023), indicating that combining dual-effect natural compounds with autophagy inhibitors holds great potential for melanoma therapy. Collectively, these findings highlight the potential of natural bioactive compounds to regulate the MAPK–autophagy axis, presenting a promising therapeutic strategy to combat skin aging and skin cancer. Further studies are needed to identify additional natural bioactive compounds targeting the MAPK–autophagy axis and to elucidate their precise mechanisms, thereby accelerating clinical translation in this field.

**TABLE 2:** MAPK–autophagy axis modulation by natural bioactive compounds in skin aging.

Classification	Bioactive compounds	Effect on MAPK	Mechanism	Consequence	References
Flavonoids	Bamboo leaf flavonoids	Inhibition p38	p-p38↓, LC3-II/I, Beclin-1↑, p21, p16, K9M-H3↓	Suppressed oxidative stress-induced senescence of HaCaT keratinocytes and UVB-induced photoaging of mice	Gu et al. (2022b)
	4,4′-Dimethoxychalcone	Inhibition p38, JNK	p-p38, p-JNK↓, p-ULK1, LC3-II/I, Beclin-1↑, p21, p16, K9M-H3↓		Gu et al. (2022a)
	Taxifolin	Activation p38, JNK, ERK1/2	p-p38, p-JNK, p-ERK1/2↑, LC3 II/I, Beclin-1, ATG5, ATG7↑, p62↓	Promoted traumatic skin repair in mice	Ding et al. (2023)
Traditional Chinese medicine	Ginsenoside Rg3	Inhibition p38, JNK, ERK	p-p38, p-JNK, p-ERK, NF-κB↓, p62↓, LC3-II/I, Beclin-1↑	Promoted skin healing in full-thickness skin defect models in mice	Peng et al. (2022)
	Resveratrol	Inhibition p38	p-p38, HIF-1α, p53↓, LC3-II, Beclin-1↓, cleaved caspase3, Bax↑	Induced transition from protective autophagy to apoptosis in human skin hypertrophic scar fibroblasts	Zuo and Ma (2024)
Nucleic acid	Exogenous nucleotides	Inhibition p38, JNK, ERK1/2	p-p38, p-JNK, p-ERK1/2↓, p-AMPK↑, p62↓, LC3-II/I↑, p16↓	Improved skin aging in senescence-accelerated mouse prone-8 mice	Fan et al. (2024)
Carotenoids	Astaxanthin	Inhibition ERK	p-ERK, p-NF-κB↓, LC3-II, Beclin-1, ATG12, ATG14↓	Inhibited Bisphenol A-induced autophagic cell death in HDFs	Lim et al. (2021)
Plant lectins	Polygonatum cyrtonema lectin	Activation p38	p-p38, p-p53↑, LC3-II/I, Beclin-1↑, Bax, Cytochrome c, Caspase-3, Caspase-9, Cleaved PARP↑, Bcl-2, Bcl-XL↓	Induced pro-apoptotic autophagy in human melanoma A375 cells	Liu et al. (2009)
Lipid Extracts	Algal oil-derived n-3 polyunsaturated fatty acids	Activation JNK1/2 Inhibition p38	p-JNK1/2, LC3-II↑, p-p38, p62, IL-1β↓	Inhibited the metastatic outgrowth of melanoma cells	Tan et al. (2018)
Amide alkaloids	Piperlongumine	Activation ERK	p-ERK↑, LC3-II↑, Cleaved PARP, Bax↑, Bcl-2↓	Induced apoptosis and protective autophagy in human melanoma A375P cells	Jeon et al. (2023)
Bufadienolides	Kalantuboside B	Activation ERK	p-ERK↑, LC3-II/I↑, Cleaved PARP↓	Induced apoptosis and protective autophagy in human melanoma A2058 cells	Hseu et al. (2019)

## 6 Conclusion

As described in this review, the MAPK–autophagy interplay critically regulates senescence in keratinocytes, fibroblasts, and melanocytes, and influences skin aging-related processes such as epidermal barrier function, wound healing, and melanoma progression. MAPKs are important mediators of autophagy upon various stresses, such as oxidative stress, UV radiation, and hypoxia. Notably, MAPK activation does not always accelerate skin aging but can instead attenuate it through autophagy induction. However, MAPK-mediated autophagy exhibits dual effects that can either inhibit senescence or contribute to senescent cell accumulation and tumor drug resistance progression. Autophagy can also affect MAPK signaling activity to modulate skin aging. Therefore, the regulatory role of the MAPK–autophagy interplay is highly context-dependent, with its functional outcomes influenced by the intervention strategy, stress condition, cell model (e.g., cell type or origin), and the specific MAPK subtype involved. Future studies should precisely target the MAPK–autophagy interplay and elucidate the exact mechanisms underlying its multifaceted roles by strictly controlling these influencing factors. Such efforts will bridge the current gap in clinical translation and provide innovative strategies for developing effective skin anti-aging therapies.

## 7 Future perspectives

Although natural bioactive compounds demonstrate anti-aging effects via MAPK–autophagy axis modulation in preclinical studies, clinical applications targeting this axis remain unexplored. Several natural compounds, such as astaxanthin (Ito et al., 2018; Phetcharat et al., 2015; Tominaga et al., 2012; Yoon et al., 2014), resveratrol (Maruki-Uchida et al., 2018; Shi et al., 2024), taxifolin (Micek et al., 2021), and polyunsaturated fatty acids (McDaniel et al., 2008; Pilkington et al., 2013), have shown clinically measurable anti-aging effects, providing strong evidence for the feasibility of this therapeutic strategy. To bridge the preclinical-clinical divide, the implementation of skin organoid models emerges as a promising translational strategy (Hong et al., 2023; Wang et al., 2024). By integrating advanced 3D culture techniques (e.g., 3D bioprinting) (Cubo et al., 2016), genetic engineering tools (e.g., CRISPR-Cas9) (Hendriks et al., 2020), and artificial intelligence and machine learning approaches (Flament et al., 2022; Gantenbein et al., 2025; Xiao et al., 2023), these models can better recapitulate skin aging and age-related skin cancer progression. This integrated approach will not only elucidate the anti-aging regulatory mechanisms of natural compounds targeting the MAPK–autophagy axis across diverse physiological and pathological contexts but also accelerate the discovery and screening of novel bioactive compounds from natural sources. Accurate and efficient delivery system is also a challenge that cannot be ignored. Nanocarriers and hydrogels have shown therapeutic potential (Ding et al., 2023; Peng et al., 2022; Zuo and Ma, 2024). An innovative approach is to integrate them with physical drug delivery enhancement techniques, such as electrical (e.g., iontophoresis), mechanical (e.g., microneedles), sound (e.g., sonophoresis) and thermal (e.g., fractional laser) methods (Yamada and Prow, 2020), to further improve the bioavailability and therapeutic efficacy of natural active compounds.

Future studies should prioritize elucidating the dynamic interplay between MAPK and autophagy across diverse aging microenvironments, along with the precise molecular mechanisms modulating skin aging progression, to comprehensively decipher their context-dependent regulatory networks. Furthermore, in melanoma treatment, regulating autophagic flux through the MAPK pathway to mediate the switch from pro-survival to pro-death autophagy has significant therapeutic implications. Therefore, investigating the molecular markers of autophagy-associated death and defining the “tipping point” between autophagy-mediated cell protection and killing may provide novel therapeutic strategies for melanoma. In summary, the MAPK–autophagy interplay represents a promising therapeutic target for anti-skin aging interventions and melanoma treatment. Multidisciplinary collaboration is urgently needed to elucidate these mechanisms and accelerate clinical translation. Further well-designed pharmacological and clinical studies are warranted to target this dual-pathway approach and develop effective skin anti-aging strategies.

## Glossary

UV: Ultraviolet
TGF-β: Transforming growth factor-β
NF-κB: Nuclear factor kappa B
NRF2: nuclear factor erythroid 2-related factor 2
MAPK: Mitogen-activated protein kinase
ROS: Reactive oxygen species
ECM: Extracellular matrix
DEJ: Dermal–epidermal junction
MMPs: Matrix metalloproteinases
CMA: Chaperone-mediated autophagy
ER: Endoplasmic reticulum
AMPK: AMP-activated protein kinase
mTORC1: Mammalian target of rapamycin complex 1
ULK1: UNC-51 like kinase 1
ATG: Autophagy-related gene
FIP200: Focal adhesion kinase family interacting protein of 200 kDa
PI3P: Phosphatidylinositol 3-phosphate
PI3K: Phosphoinositide 3-kinase
AMBRA1: Autophagy and Beclin-1 regulator 1
UVRAG: UV radiation resistance-associated gene
Bcl-2: B-cell lymphoma-2
LC3: Microtubule-associated protein light chain 3
PE: Phosphatidylethanolamine
WIPI: WD-repeat protein interacting with phosphoinositide
HDFs: Human dermal fibroblasts
ERK: Extracellular signal-regulated kinases
JNK: c-Jun amino-terminal kinases
AP-1: Activator protein 1
TIMPs: Tissue inhibitors of metalloproteinases
TNF-α: Tumor necrosis factor-α
COX-2: Cyclooxygenase-2
HO-1: Heme oxygenase-1
K9M-H3: K9 trimethylated histone 3
BNIP3: BCL2 and adenovirus E1B 19-kDa interacting protein 3
FGFR2b: Fibroblast growth factor receptor 2b
BRAF: B-Raf proto-oncogene
CCL2: C-C motif chemokine ligand 2
AGEs: Advanced glycation end products
HIF-1α: Hypoxia-inducible factor-1α
MITF: Microphthalmia transcription factor
α-MSH: α-melanocyte-stimulating hormone
CREB: cAMP response element-binding protein
TYR: Tyrosinase
PMEL 17: Premelanosome protein 17
TRP: Tyrosinase-related proteins
Bcl-XL: B-cell lymphoma-extra large
PERK: Protein kinase R-like endoplasmic reticulum kinase
ATF4: Activating transcription factor 4
